# Brain Tumour Segmentation and Grading Using Local and Global Context-Aggregated Attention Network Architecture

**DOI:** 10.3390/bioengineering12050552

**Published:** 2025-05-21

**Authors:** Ahmed Abdulhakim Al-Absi, Rui Fu, Nadhem Ebrahim, Mohammed Abdulhakim Al-Absi, Dae-Ki Kang

**Affiliations:** 1 Department of Smart Computing, Kyungdong University, 46 4-gil, Bongpo, Gosung 24764, Republic of Korea; absiahmed@kduniv.ac.kr (A.A.A.-A.); mohammed@kduniv.ac.kr (M.A.A.-A.); 2 College of Language Intelligence, Language & Brain Research Center, Sichuan International Studies University, Chongqing 400031, China; furui19891209@wfust.edu.cn; 3 Department of Computer Science, College of Engineering and Polymer Science, University of Akron Ohio, Akron, OH 44325, USA; nebrahim@uakron.edu; 4 Department of Computer & Information Engineering, Dongseo University, 47 Jurye-ro, Sasang-gu, Busan 47011, Republic of Korea

**Keywords:** MRI, brain tumour, glioma detection, segmentation, classification

## Abstract

Brain tumours (BTs) are among the most dangerous and life-threatening cancers in humans of all ages, and the early detection of BTs can make a huge difference to their treatment. However, grade recognition is a challenging issue for radiologists involved in automated diagnosis and healthcare monitoring. Recent research has been motivated by the search for deep learning-based mechanisms for segmentation and grading to assist radiologists in diagnostic analysis. Segmentation refers to the identification and delineation of tumour regions in medical images, while classification classifies based on tumour characteristics, such as the size, location and enhancement pattern. The main aim of this research is to design and develop an intelligent model that can detect and grade tumours more effectively. This research develops an aggregated architecture called LGCNet, which combines a local context attention network and a global context attention network. LGCNet makes use of information extracted through the task, dimension and scale. Specifically, a global context attention network is developed for capturing multiple-scale features, and a local context attention network is designed for specific tasks. Thereafter, both networks are aggregated, and the learning network is designed to balance all the tasks by combining the loss functions of the classification and segmentation. The main advantage of LGCNet is its dedicated network for a specific task. The proposed model is evaluated by considering the BraTS2019 dataset with different metrics, such as the Dice score, sensitivity, specificity and Hausdorff score. Comparative analysis with the existing model shows marginal improvement and provides scope for further research into BT segmentation and classification. The scope of this study focuses on the BraTS2019 dataset, with future work aiming to extend the applicability of the model to different clinical and imaging environments.

## 1. Introduction

The human brain is a rigid and volume-restricted part of the body; thus, human capacity might be influenced through unforeseen development. The unnatural and uncontrolled development of brain cells is known as a brain tumour (BT). Brain tumours are those whose initial source includes the brain. In addition, brain tumours may proliferate into other organs in the body, resulting in life-threatening conditions. Moreover, according to the World Health Organization, BTs account for 2% of all human cancers. In general, BTs are classified based on the type and severity of benign and malignant tumours [1,2,3]. Gliomas are considered primary malignancies of the central nervous system (CNS). In general, gliomas are classified as fast progressive lesions (Grade 3 and Grade 4) and slow progressive lesions (Grade 1 and Grade 2). Moreover, Grade 1 and Grade 2 are categorized as low-grade gliomas (LGG), whereas Grade 3 and Grade 4 are categorized as high-grade gliomas (HGG). Glioma tumours occur in progenitor or glial cells, and they account for 26.7% of CNS and primary brain tumours [4]. Gliomas are mainly found in the temporal, frontal, and parietal lobes of the brain but rarely occur in the occipital lobe; they may also develop in the cerebellum, cauda equina, and spinal cord.

**Figure 1:**
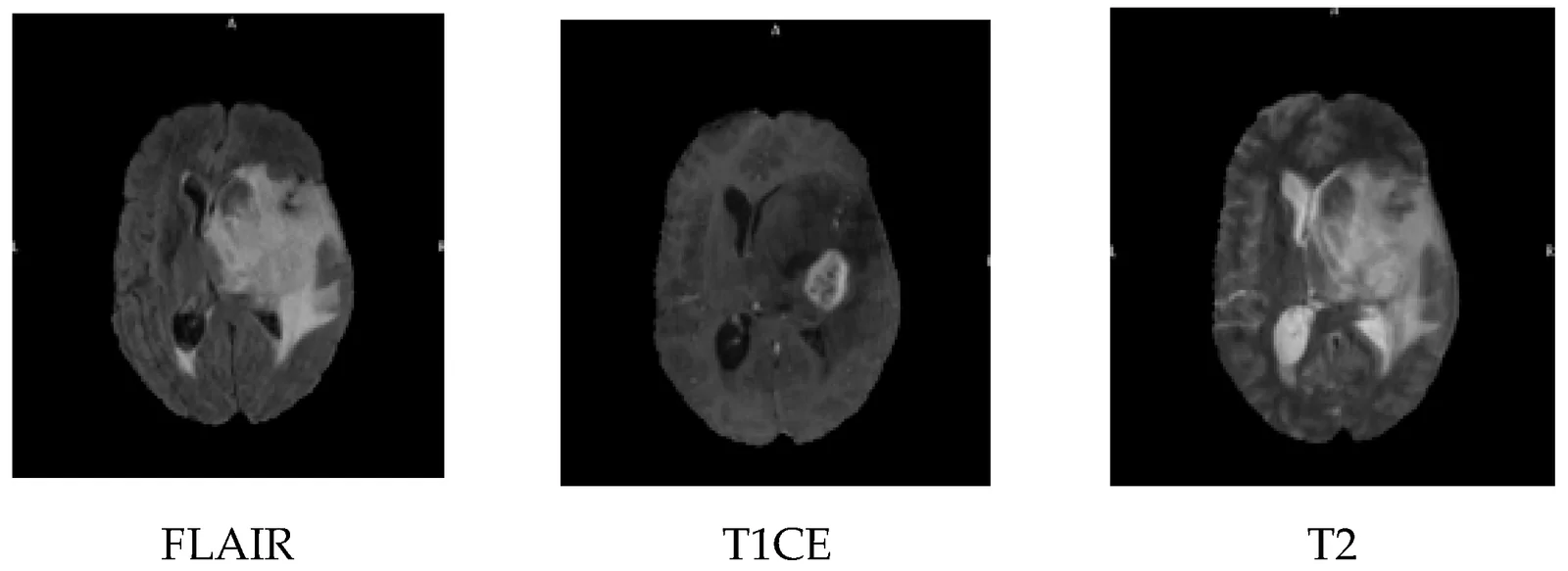
Magnetic resonance imaging sequences of the brain (left to right): FLAIR, T1CE, and T2 sequences.

Radiological images are non-intrusive methods that avoid the use of ionizing radiation. Magnetic resonance imaging (MRI) is one of the most popular neuroimaging tools, as it provides three-dimensional images of the brain with excellent resolution and contrast [5]. MRI imaging is carried out in three planes, the sagittal, coronal and axial; the MRI sequences include contrast-enhanced fluid automation inversion recovery (FLAIR), T1-weighted with contrast enhancement (T1CE), and T2-weighted images, as shown in Figure 1. FLAIR is an MRI sequence that suppresses the signal from cerebrospinal fluid, making lesions more visible, especially in periventricular areas. T1CE imaging involves the use of a contrast agent, typically gadolinium, to highlight areas with a disrupted blood-brain barrier, such as active tumour regions. T2-weighted imaging provides high sensitivity to water content and is effective in identifying oedema and tumour-related swelling.

A major obstacle in MRI segmentation is the presence of visual impressions of the skin, eyeballs and other non-brain tissues, and the visual representations of these tissues must be stripped to obtain clean images. Several methods, including atlas-based, hybrid-based, intensity-based and morphology-based mechanisms, are utilized for cleaning the MRI images. 

Despite many research in brain tumour’s classifications, precisely it is hard to distinguish between low-grade and high-grade gliomas, and it remains a significant challenge due to the overlapping of their visual characteristics and intratumoral heterogeneity. Current methods usually rely on handcrafted features or shallow learning models, which limits the classification accuracy, and generalizing across a dataset, many existing approaches do not fully exploit deep semantic features or multi-scale contextual information from MRI data [6,7]. The most existing models rely on a single shared network for both segmentation and classification, which leads to suboptimal performance. Our proposed LGCNet addresses these limitations by introducing task-specific attention networks (local and global), allowing for more accurate and robust brain tumour segmentation and grading. The deep learning-based LGCNet aims to enhance the tumour classification performance by automatically learning rich, hierarchical features from imaging data without the need for extensive manual intervention. The proposed method is systematically compared with the existing state-of-the-art techniques, which demonstrates its effectiveness in improving the glioma grade classification accuracy and robustness.

**Figure 2:**
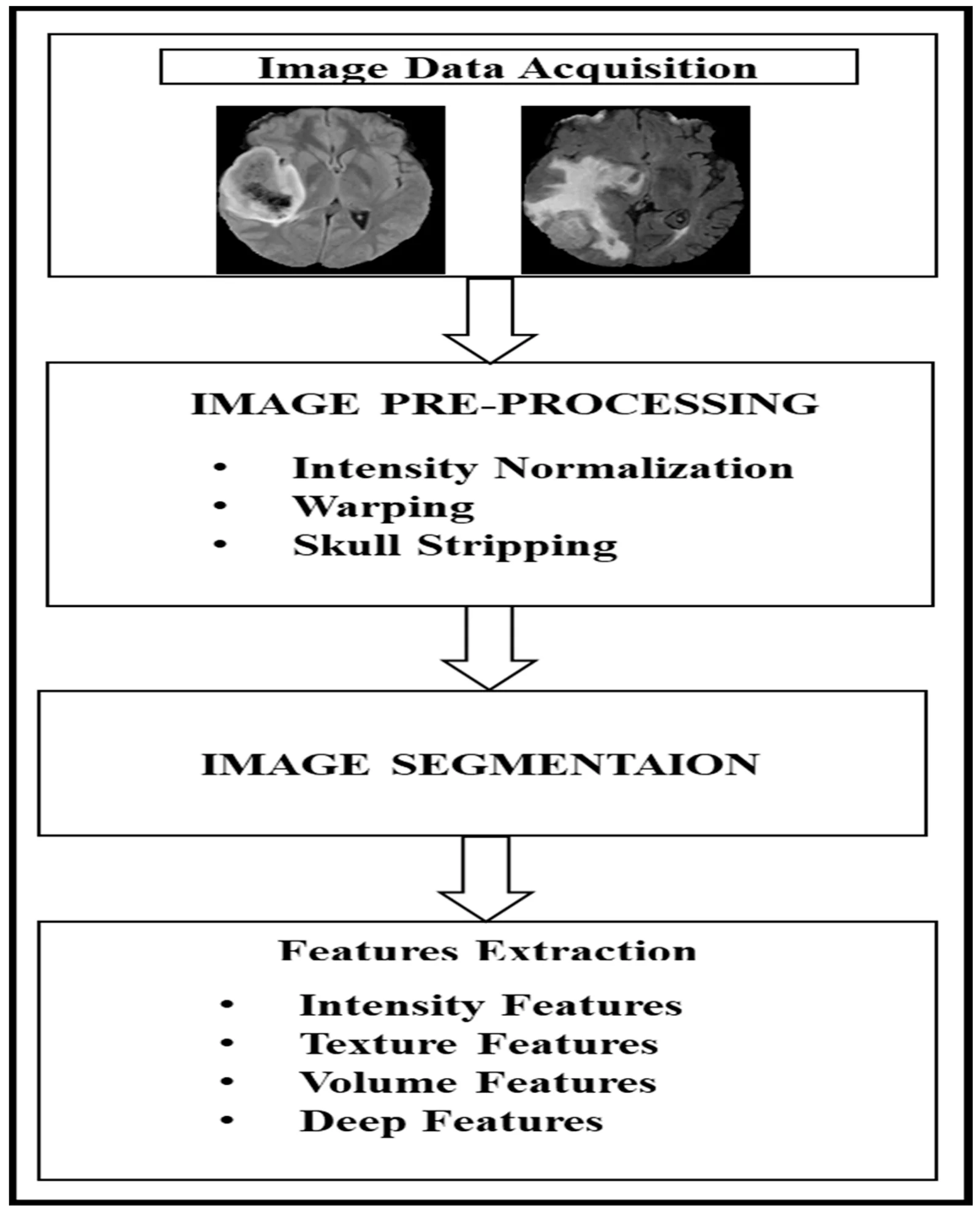
General process of radionics extraction.

Early detection and classification are of utmost importance for the effective and timely treatment of BTs. As the human visual cortex (HVC) has restricted capability for deciding the grade level of lesions via MRI, computer-aided diagnosis (CAD) models were developed to support radiologists in visualising and defining the types of tumours. These automated approaches include tumour detection, segmentation and classification. Radiomics is another quantitative approach for the extraction of a large number of features from medical images [8]. Figure 2 shows the general process of radiomics extraction for MRI sequences, which comprises four steps. The first step is image data acquisition through MRI scanning, and the second step is image pre-processing (which includes intensity normalization, warping, and skull stripping). The third step is image segmentation with various sub-area segmentation, and the fourth step involves feature extraction (which includes various image-related features). Image processing and computer vision have been able to provide an efficient mechanism for the automated detection, segmentation and classification of features. However, existing radiomics-based mechanisms tend to ignore the peritumoral environment and focus solely on intratumoral features in grading the glioma [3].

Automated approaches for segmentation are classified into two broad categories: traditional machine learning (ML) approaches and deep learning-based approaches. Traditional ML approaches mainly rely on low-level (LL) features [9,10]. Segmentation is one of the major processes used in the detection of a tumour, as it highlights the region of interest (ROI) and others that are similarly detected are selected for classification or grading.

In the traditional ML approach, the segmentation includes the estimation of the tumour boundaries. Recently, deep learning has been adopted in biomedical image analysis and computer vision for the improvement of feature extraction from images through an automated approach. Deep learning relies on the training data and eliminates the major pre-processing required for traditional ML. The deep learning-based model utilizes a convolutional neural network (CNN) with three steps. The first is pre-processing, which includes discarding noise along with segmentation. The second step is training, where the learned features and labels of an individual image are given to the classifier for training [11,12,13,14] and where the classifier learns to identify the various grades or classes from the training data. The third step is the testing phase, which includes the same feature extraction process that is used for training purposes, but it is used for extracting the features from a single query image. Later, a feature vector is passed to the trained classifier to predict the grade of the tumour. The deep learning-based approach achieves better metrics than those for other techniques, which makes it more suitable for radiologists to use for real-time application in a clinical setting [15,16,17]. Artificial intelligence (AI) could be a boon for patient management related to cancer, as it enables the early detection of gliomas and can be used for determining the prognosis for a patient. Current exploration regarding early detection and grading has not been feasible for implementation in the clinical management of gliomas. Our motivation is to explore recent developments in deep learning for early detection and survival prediction; we also aim to identify research gaps. Thus, motivated by the exploration of medical image analysis and artificial intelligence, this research develops a deep learning-based model in which a local and global context-aware aggregation network (LGCNet) is implemented for predicting and classifying the BT in a given image. Further contributions of this research are highlighted as follows:

- LGCNet comprises two dedicated networks. The local context attention (LCANet) is designed for exploiting the local features on the task-specific requirement, and the global context attention network (GCANet) is designed for extracting the global features.
- The local context attention network yields particular task-specific features. GCANet exploits the relatedness among the tasks to achieving robust feature representation through a bidirectional layer and spatial attention layer. GCANet is introduced to perform the weighted feature fusion by dynamically capturing inter-task dependencies and enhancing global context representation.
- LCA-Net is proposed for utilizing the soft attention mask that combines the segmentation and classification losses for the absolute inference of a particular task.
- The proposed model is evaluated considering the BraTS 2019 challenge dataset for segmentation and grading, which considers metrics such as the Dice score, sensitivity, specificity, and Hausdorff distance. A comparative analysis is carried out with the proposed model against the other deep learning-based models to prove the efficiency of the proposed model.

Deep learning enhances our ability to analyse brain tumours, but many models use a single network for both segmentation and classification, which struggles to produce accurate results. These models often miss local details and the overall context of the tumour. To address this problem, we propose LGCNet. It uses two independent networks: one focused on local features and another focused on global features. This design allows the model to better segment and classify gliomas of varying shapes and sizes.

The LGCNet model employs LCANet and GCANet mechanisms to enhance its performance in terms of brain tumour segmentation and grading. LCANet focuses on capturing detailed, task-specific features within localized regions of the image, which is crucial for identifying subtle details and fine-grained structures. For example, in cases of small or less prominent tumours, LCANet enhances the ability of the model to detect subtle anomalies by emphasizing local features that may indicate the presence of a tumour. In contrast, GCANet captures broader contextual information that is essential for understanding the overall structure and spatial relationships within the image. This is particularly important for detecting large or diffuse tumours, where the global context aids in delineating the tumour boundaries more accurately. By combining local and global attention mechanisms, LGCNet effectively integrates fine details with contextual information, leading to improved segmentation accuracy and robustness across different tumour grades and sizes. This dual approach yields a model having better generalization to varied datasets, as the model can adapt to different imaging conditions and tumour characteristics more effectively.

This article is organized as follows. The first section includes background information on BTs, MRI, and research in the detection of BTs, along with the motivation for and contribution of this work. The second section discusses the relevant related work, including the methodologies and shortcomings of prior studies. The third section describes the mathematical formulation and architecture of the proposed LGCNet, and the fourth section includes an evaluation of LGCNet by considering different metrics. Finally, some conclusions based on the findings of this study are presented, along with the limitations of this study and some possible avenues for future work.

## 2. Related Work

The popularity of traditional machine learning techniques and unsupervised techniques has waned in the last few years, with research trends leaning toward the use of efficient deep networks [17], and this section focuses on relevant deep learning-based techniques for brain tumour detection and classification. In the CAD model presented by Wang et al. [18], BT MRI results are segmented using a convolution operation fused with principal component analysis to accomplish the feature extraction. A performance analysis indicated the early improvement with limited data. Zhu et al. [19] explored the potential of deep learning in extracting the total extraocular muscles and optic nerves from orbital computed tomography (CT) images, highlighting the capability of semantic segmentation models to handle complex anatomical structures. While this approach achieves high precision and is tailored to orbital CT scans, it may not be as effective for other types of medical images due to its specialized nature. In contrast, our method employs a more generalized approach that adapts seamlessly across different imaging techniques, offering broader applicability without sacrificing accuracy.

Liu et al. [20] introduced a transformer and a convolutional-based dual branch network for retinal vessel segmentation in optical coherence tomography angiography images, demonstrating how combining these two powerful architectures can enhance the capture of detailed features. Their model effectively balances local and global contextual information, which is crucial for detailed vascular imaging. Our model similarly integrates multiple architectural features but focuses on reducing the computational demands to facilitate quicker processing times, which is essential for clinical applications. Mu et al. [21] presented an innovative approach using an attention-augmented residual U-Net for vasculature segmentation, including differential pre-processing and geometric post-processing to enhance the detection of intracranial aneurysms. Their method is particularly adept at segmenting intricate vascular networks and pathological features. Unlike the approach used by Mu et al., our approach simplifies the segmentation process by eliminating the need for extensive pre-processing and post-processing, thus streamlining the workflow for medical practitioners and reducing the time to diagnosis.

Özyurt et al. [22] studied tumour recognition along with classification using the applied fuzzy c means algorithm and CNN architecture (named SqueezeNet) that uses an extreme learning machine algorithm for classification. This model achieves a 10% improvement over other techniques. Another CNN model was used in Çinar and Yildirim [23] for BT identification, and the ResNet50 residual network with 10 extra layers was integrated for better performance and to achieve better metrics than the existing ResNet architecture. An attention-based CNN architecture called BrainMRNet was proposed by Togaçar et al. [24] for brain tumour detection. Moreover, the attention module along with the hypercolumn mechanism aids in exploiting the optimal features from the ROI. BrainMRI achieves better metrics than VGG-16, AlexNet, and GoogleNet when using the same dataset. In Saba et al. [25], deep features were acquired using the VGG-19 architecture through a grab–cut segmentation mechanism along with a handcrafted approach. Optimized features are integrated into one feature vector prior given to the different classifiers for healthy image and glioma detection. Cheng et al. [26] developed a deep network named Multi-Modal Variation AE (MMD-VAE) for grading gliomas based on the radionics features. Here, the quantization of the radiomic features is carried out from the ROI in the case of each modality; further latent representations of the variational autoencoder are extracted to obtain the complementary data among the modalities. Furthermore, cross-modality reconstruction is adopted for effective implementation. Liu et al. [27] developed a CANet Model for glioma segmentation that aims to capture high-dimensional features with context from conditional random fields and convolutional space. Afterwards, context-guided attentive conditional random fields are employed to aggregate the features. Cheng et al. [28] developed a multi-tasking learning model for simultaneous IDH genotyping and glioma segmentation, in which the heterogeneity and task correlation are solved with an integrated CNN transformer encoder, which comprises a transformer and a CNN for extracting the global information and spatial information. Afterwards, the loss function is designed for balancing the two tasks (i.e., segmentation and classification), and semi-supervised learning is used for accuracy improvisation. An AI-based radiomic analysis mechanism for slice pooling, developed by Zhao et al. [29] and referred to as AI-RASP, generates compressed images through grey value compression of each MRI slice for radiologists to use for the manual segmentation of the images. AI-RASP concatenates the radiomics model for the verification of the glioma grading effect and aims to reduce the time required for segmentation. Tupe-Waghmare et al. [30] built a multi-task model based on a semi-supervised approach that incorporates unlabelled glioma data to obtain multiple molecular predictions. Furthermore, this model employs the latent space through the use of an encoder. Furthermore, Cheng et al. [31] introduced an algorithm for capturing the peritumoral region of the glioma with a particular radius; moreover, 285 patients were scanned, and a total of 2153 radiomic features through the peritumoral volumes and intratumoral volumes through mpMRI scans were further refined using a feature-ranking technique. The top-ranking features were fed to the classifiers for glioma prediction. Xiao et al. [32] designed a novel neural network architecture called DLS-DARTS with two learnable stems to fuse multimodal low-level features, and they utilized a derivation approach to improve the accuracy and area under the curve.

In addition to applications in medical imaging, decision intelligence frameworks have also shown promising results in other technical areas. For example, Sattar et al. [33] proposed a prediction model using K-Nearest Neighbour (KNN) in combination with Grey Wolf Optimization to evaluate the stability of hard rock pillars in mining, demonstrating the effective integration of traditional ML with metaheuristic optimization techniques. Similarly, Azamathulla et al. [34] developed a multi-criteria decision intelligence framework for predicting fire risk in underground structures. Both research studies highlight the growing potential of hybrid and intelligent systems for high-risk decision making, which is consistent with the goals of our model in the field of medical diagnostics.

Considering the development of BT detection and classification, a brief survey of the deep learning-based mechanisms suggests that it can accurately assist radiologists in the prediction of the tumour region as well as in classification. However, there are various challenges that remain, as most deep learning models with a single network for all tasks are less impactful. Thus, this research work develops LGCNet, which is a dedicated network for specific tasks.

To clearly summarize the models discussed in the relevant existing works, Table 1 compares their main features, advantages, and limitations.

## 3. Proposed Methodology

Despite the remarkable performance of deep learning mechanisms for accomplishing various tasks, the majority of cutting-edge techniques rely on large-scale annotated training data that are unavailable for healthcare and clinical tasks, and the cost for labelling during medical image segmentation is high because it requires effort by a person with the appropriate expertise. Thus, to grade a brain image, automatic segmentation plays a major role. This section presents the architecture of the proposed LGCNet model. The proposed model adopts the attention mechanism, as it effectively solves the issue of convolution operation, which cannot focus on target features; in addition, it can be used for optimizing the noise in a hidden layer of a network. For this reason, it has become one of the trending research options for a task-specific model.

The execution time for the LGCNet model is crucial for its practicality in clinical settings. Training the model, which involves complex convolutional layers and attention mechanisms, requires significant computational resources, typically taking from several hours to days to accomplish on our Tesla P100 GPU with 16 GB of RAM. However, the inference phase—in which the model segments and grades new MRI images—is much faster, often processing scans in seconds to a few minutes. This quick inference time makes the LGCNet feasible for real-time clinical applications, allowing radiologists to promptly and accurately diagnose tumours and plan treatments. Thus, despite the intensive training phase, the model’s efficient inference ensures its practicality and effectiveness in clinical settings.

**Figure 3:**
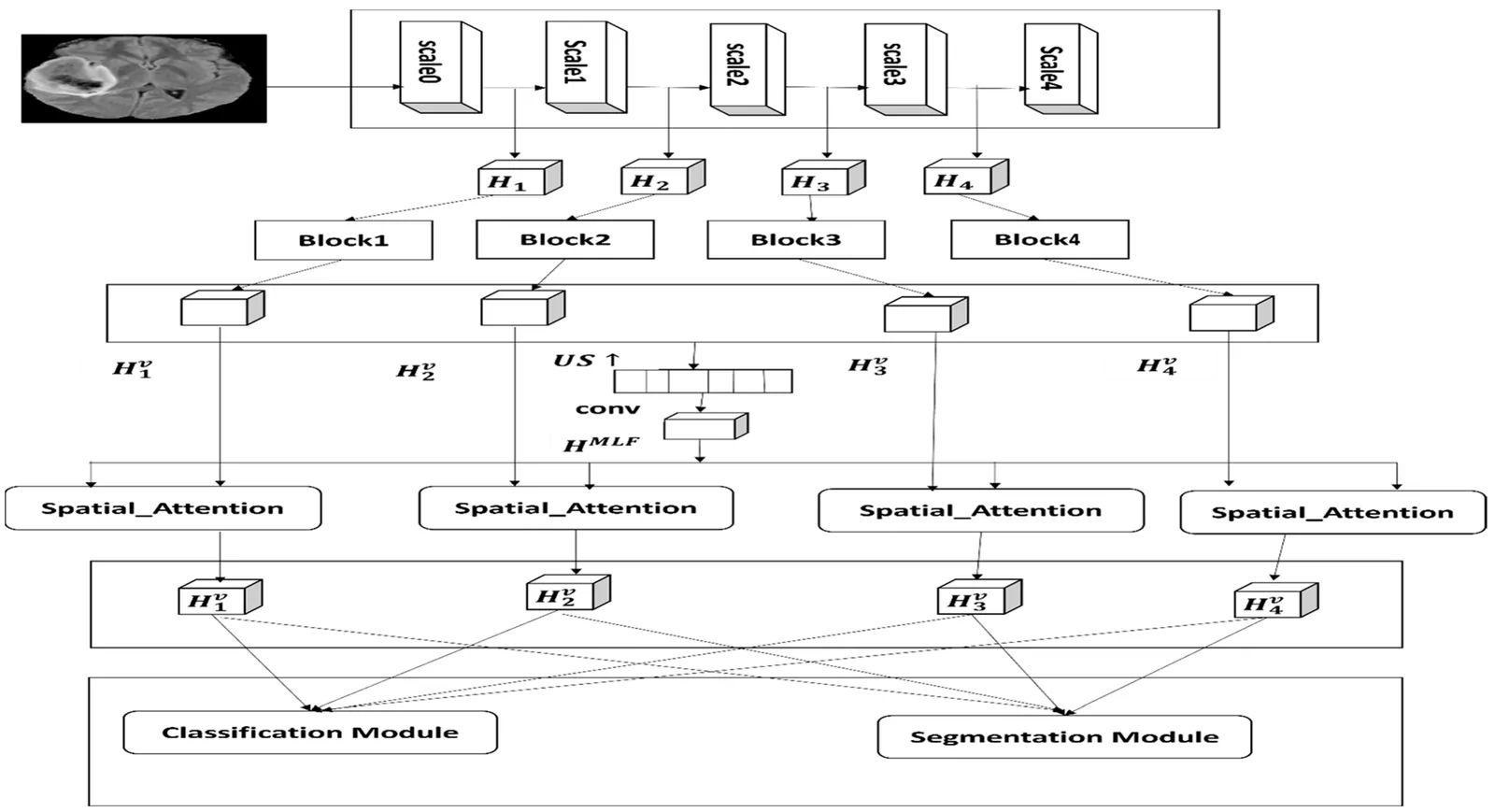
LGCNet Framework.

Figure 3 shows the proposed architecture that includes three parts—the backbone, the global context attention network (GCANet) and the local context attention network (LCANet)—where both networks are designed for feature learning. The LGCNet framework includes a local attention network along with a deep supervision path and a global attention network, and it shows four blocks, where the blocks represents the visual_attention block, spatial attention; $H_{1}$, $H_{2}$, $H_{3}$ and $H_{4}$ present multi-label features; $H_{1}^{X}$, $H_{2}^{X}$, $H_{3}^{X}$ and $H_{4}^{X}$ represent multi-label features along with higher consistency; and $H_{1}^{C}$, $H_{2}^{C}$, $H_{3}^{C}$ and $H_{4}^{C}$ indicate the optimized and global multi-label features.

The proposed architecture takes the MRI sequence as input and adopts a custom CNN as the backbone, inspired by the work of Wang et al. [35], for acquiring the multi-label features at different resolutions, as there are two different feature maps (large-scale maps and low-scale maps). Large-scale feature maps have a high resolution with rich information regarding the image, whereas low-scale feature maps have a low resolution with high semantic information. Hence, multi-label features act effectively with various tumour sizes. Thereafter, considering the nature of image-based research, a computer with a huge amount of memory is required; thus, Scale 0 has not been refined. In addition, considering the characteristics of the dataset adopted for this research, each volume contains a few slices of the target; for this reason, down-sampling of blocks in Scale 0, Scale 1 and Scale 2 is carried out by selecting the stride of (1,2,2). Afterwards, by deepening the network layer, the proposed approach adopts dilated convolution between Scale 3 and Scale 4 for aggregating multi-label semantic information and later extracting feature maps with the optimal resolution. Thereafter, the multi-label features are given as the input to scale the aware network that retains the detailed information of the targets and suppresses the noise. The refined multi-label features are considered for the input for task-aware feature learning.

**Figure 4:**
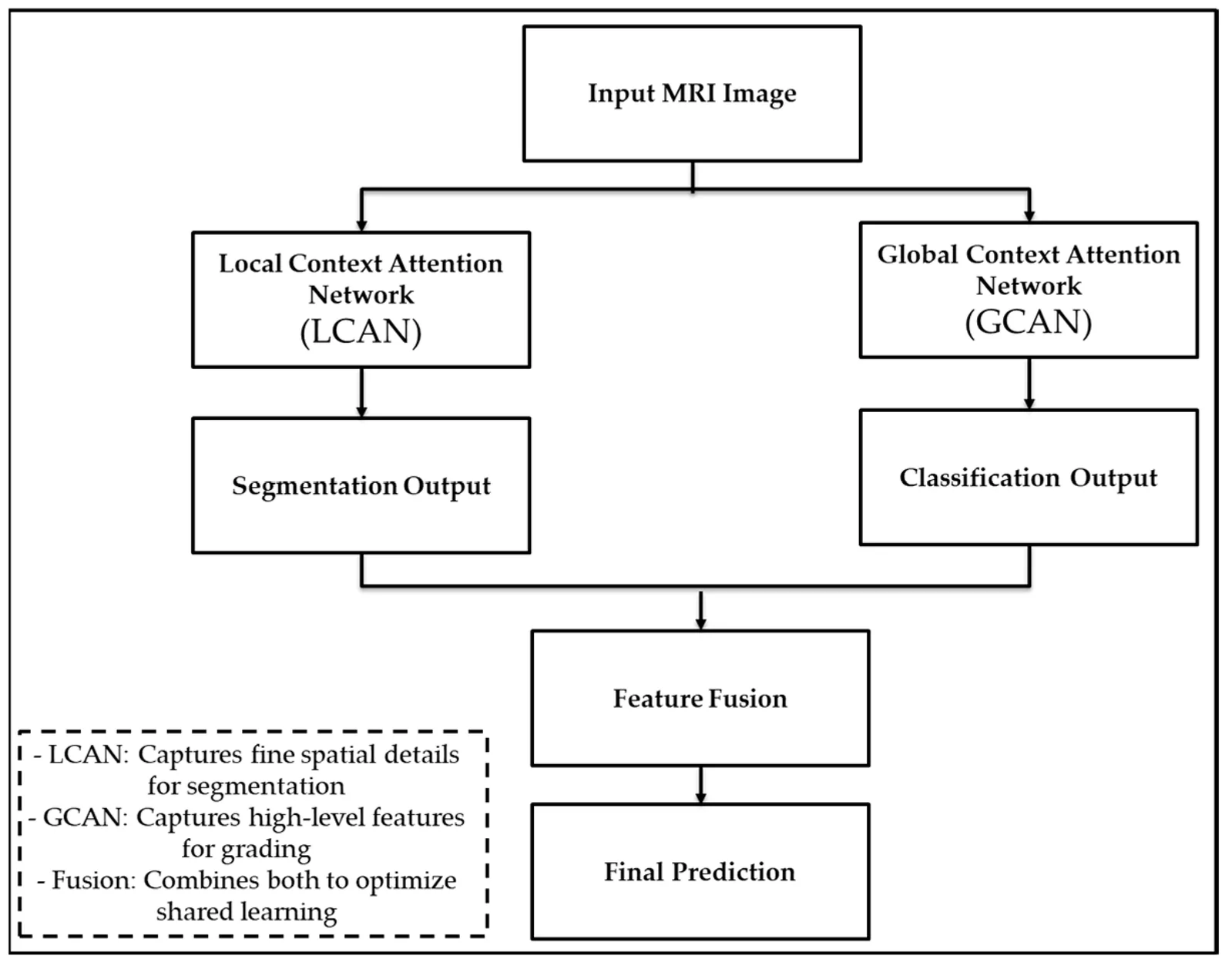
LGCNet architecture.

The LGCNet consists of two independent branches operating in parallel. The first is called LCAN, which focuses on learning detailed features of tumour regions to improve segmentation. The second is GCAN, which looks at the entire image to understand the overall tumour patterns and aid in classification. The outputs of the two branches are combined and used to train a model to perform both tasks. This structure allows LGCNet to learn both local details and the global context simultaneously, achieving higher accuracy than using a single network. Figure 4 shows that a local contextual attention network (LCAN) and a global contextual attention network (GCAN), followed by the task-specific output and feature fusion.

### 3.1. Global Context Attention Network

**Figure 5:**
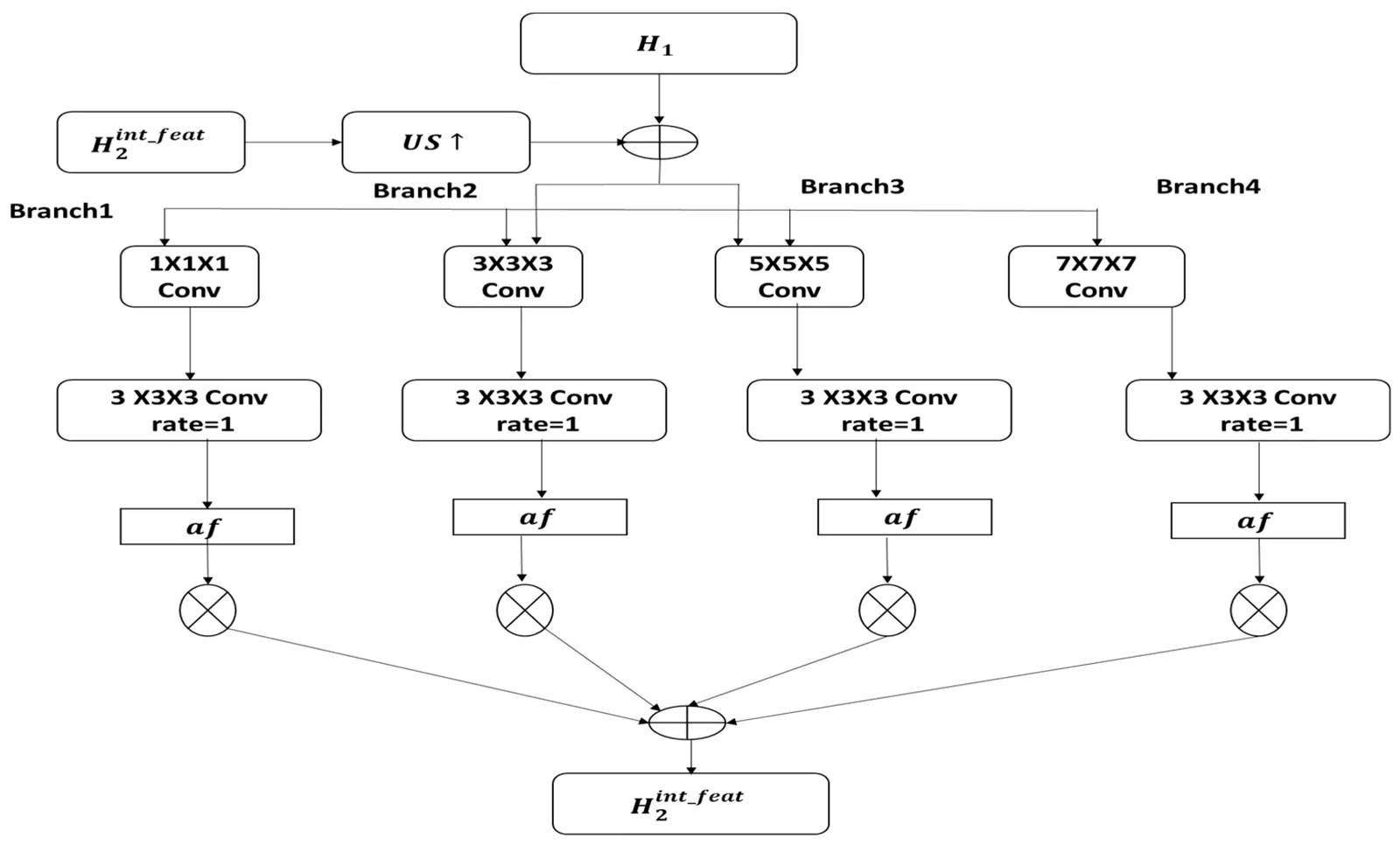
Proposed visual attention architecture.

A scale-aware network in the model is used to capture the boundary information, regional semantics and effective context information. The global context attention network (GCANet) adopts two types of feature learning: visual attention and spatial attention. Visual attention exploits the global and local information in various scale features and is adopted from Cong et al. [36]. Thereafter, an attention mechanism is introduced for the visual receptive field; in addition, various branches following scales are designed for enhancing the receptive field. In the visual attention block represented in Figure 5, the centre of vision is represented through a convolutional layer. Thereafter, these visual attention blocks form a bidirectional approach. Figure 5 shows the proposed visual attention architecture.

Figure 4 shows the architecture for visual attention block 1, which includes multiple branch features with distinctive receptive fields. Later, the bottom-up and top-down features are fused to integrate the contextual information. The visual attention block comprises multi-label feature details generated through a backbone, in which $H_{u}$ is refined and presented as $H_{u}^{x}$ and the visual perception is computed using the following equations:

(1) $$H_{u}^{int\_feat}=\left\{\begin{array}{ll} h_{u}\left(H_{u}\right) & u=4\\ h_{u}\left(\frac{\delta_{1}\cdot H_{u}+\lambda_{2}\cdot US↑\left(H_{u+1}^{int\_feat}\right)}{\delta_{1}+\delta_{2}}\right) & u\neq 4 \end{array}\right.$$

where $h_{u}$ denotes the soft-attention block within the visual attention mechanism and $H_{u}$ represents the high-dimensional feature map from the convolutional layer.

(2) $$H_{u}^{x}=\left\{\begin{array}{ll} \left(\frac{\delta_{1}^{\prime }\cdot F_{s}+\delta_{1}^{\prime }\cdot F_{s}}{\delta_{1}^{\prime }+\delta_{2}^{\prime }}\right) & u=4\\ f_{s}\left(\frac{\delta_{1}^{\prime }\cdot H_{u}+\delta_{1}^{\prime }\cdot H_{u}^{int\_feat}+\delta_{3}^{\prime }\cdot DS↓\left(H_{u-1}^{x}\right)}{\delta_{1}^{\prime }+\delta_{2}^{\prime }+\delta_{3}^{\prime }}\right) & u\neq 4 \end{array}\right.$$

where $\delta_{1}^{\prime }$ and $\delta_{2}^{\prime }$ are the scaling factors used for adjusting the feature strength, and $F_{s}$ stands for the feature space encompassing the input attributes from the previous layers. In the above equation $H_{u}^{int\_feat}$ is the last recent feature extracted through a defined top-down approach at a given scale; $h_{u}$ denotes the soft-attention block in visual_attention; $\delta_{k}$ presents the weights obtained through the activation function; US↑ represents the up-sampling and DS↓ represents the down-sampling; and a normalization function is utilized for fusing the features.

**Figure 6:**
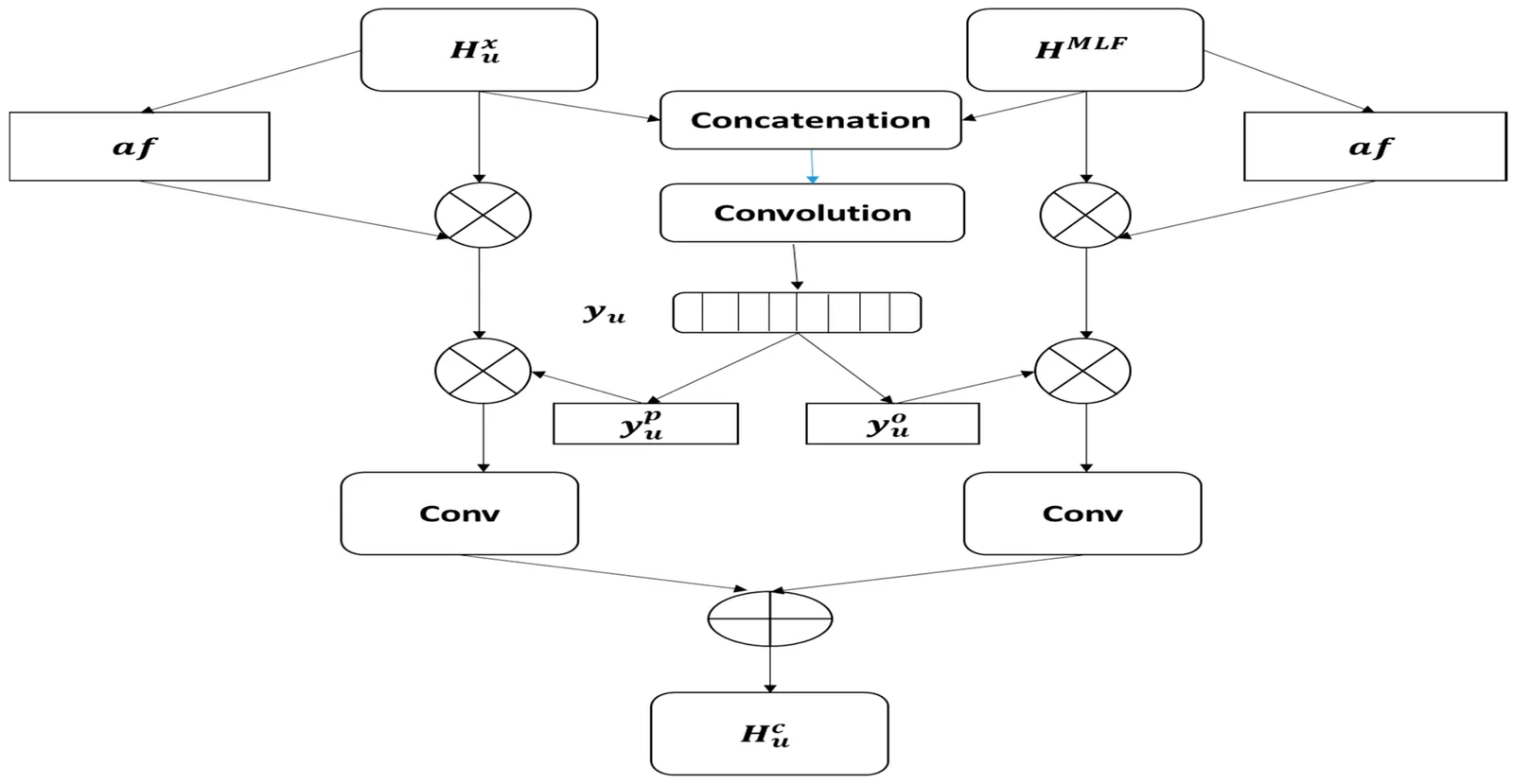
Proposed spatial attention module.

Afterwards, parameter reduction of the visual_attention block is carried out by selecting the optimal convolution; thus, the proposed visual_attention module exploits and extracts the optimal feature that has higher feature invariance. Once high feature consistency is achieved through the visual attention module, the scale_attention module shown in Figure 6, is introduced to allow the zooming of other single-scale features to Scale 1 through interpolation. Later, multi-label features are generated using the concatenation and convolution mechanism.

Furthermore, $h_{operation}$ operation is initialized to match the multi-label and single-label feature to further generate two custom feature maps using the activation function parallel. Moreover, the proposed work uses $H^{MLF}$ (a multi-label feature) and $H_{u}^{x}\;$(a single-label feature map) for learning the weight factors $y_{u}^{o}$ and $y_{s}^{p}$ using a softmax function and convolution operation. Afterwards, the soft attention feature maps and weight factors are multiplied element-wise to achieve the deep features.

(3) $$y_{u}=E\left(e\left(H_{u}^{x}\right);e(h_{operation}(H^{MLF}))\right)$$

where E denotes the operation of concatenation of the features; *e* represents a convolution operation applied to the feature maps; $H_{u}^{x}$ is a high-dimensional feature map from the visual attention network; and $h_{operation}$ indicates a transformation operation applied to $H^{MLF}$, which alters the feature map for specific tasks.

(4) $$h_{operation}=\left\{\begin{array}{ll} DS↓ & \;u\neq 1\\ K & \;u=1 \end{array}\right.$$

where DS↓ denotes the down-sampling operation applied to the feature map to reduce its dimensionality when u is not 1 and where K is specified for the case when u=1, suggesting a different operation or transformation specific to this condition.

(5) $$y_{u}^{p}=g^{e(H_{u}^{x})}\cdot {\left(g^{e(H_{u}^{x})}+g^{e(h_{operation}\left(H^{MLF}\right))}\right)}^{-1}$$

where $g^{e}$ represents a gating function applied to the feature maps to control the information flow or to modify the feature strength.

(6) $$y_{u}^{o}=g^{e(H_{u}^{x})}\cdot {\left(g^{e(H_{u}^{x})}+f^{d(h_{operation}\left(H^{O}\right))}\right)}^{-1}$$

(7) $$H_{u}^{C}=y_{u}^{p}\cdot H_{u}^{x}⊕\left(H_{u}^{x}\right)⊕y_{u}^{p}\cdot h_{operation}(H^{MLF})⊕\varphi (h_{operation}(H^{MLF})$$

Moreover, two distinctive multilevel paths are introduced to guide the extracted features.

### 3.2. Local Attention Network Modelling

The extracted deep features obtained through the model are better utilized through the design of a particular task-aware model. Unlike the previous model, our model uses a distributed approach for the segmentation and classification of tumours.

The segmentation task in the proposed work achieves a better prediction of the tumour volume through aggregating the adjacent scale features. Here, the features between the two adjacent scales are combined in a top-down way and an attention mask is utilized for guiding the specific lesion feature expression on various scales. Afterwards, high-level information is transferred to a large-scale feature.

### 3.3. Classification Module

**Figure 7:**
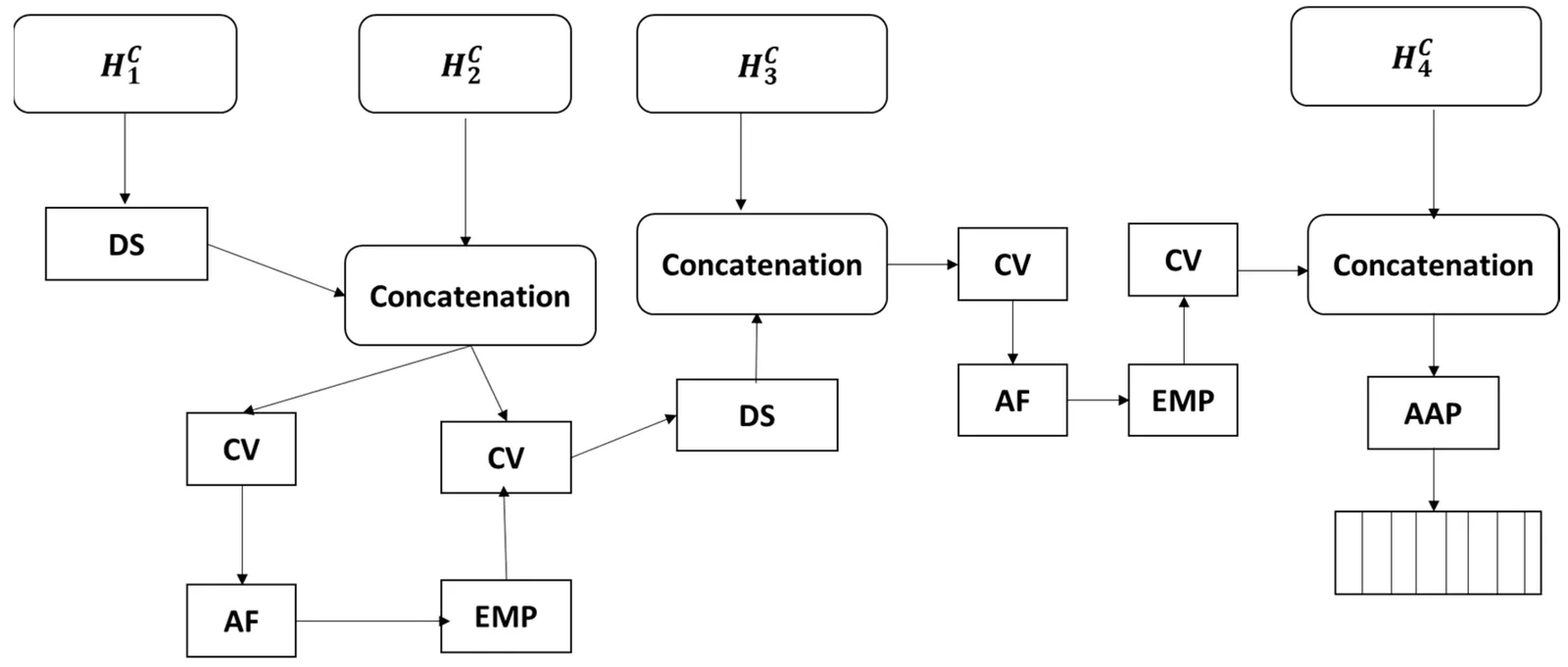
Classification module.

The classification module can predict the grading of glioma into categories (LGG or HGG) by aggregating the neighbour scale features, as in Figure 7. At first, the LGCNet model combines the features among the neighbouring scales through a bottom-up approach and then a further attention mask is used for guiding the features. Let us consider deep feature $H_{u}^{C}$ (where u∈{1,2,3,4}) and deep features with attention $H_{u}^{V}$ (where u∈{2,3}) is given as:

(8) $$H_{2}^{V}=h_{t}\left(h_{\varphi }\left(h_{\varphi^{\prime }}\left(E(H_{2}^{C};DS↓(H_{1}^{C})\right)\right)\right)⊕H_{2}^{C}$$

(9) $$H_{3}^{V}=h_{r}\left(f_{\sigma }\left(f_{r^{\prime }}\left(E(H_{2}^{C};DS↓(H_{2}^{C})\right)\right)\right)⊕H_{3}^{C}$$

where E denotes the function for the concatenation process ⊕ and indicates the multiplication; while $h_{t}$,$\;h_{\varphi }$ and $h_{t^{\prime }}$ are convolution layers along with non-linear activation and group normalization.

### 3.4. Optimization of Loss for Different Tasks

The proposed model focuses on designing the loss function for the different tasks. In addition, a hybrid loss function is computed for the segmentation approach that comprises a combination of two sum functions. First, the loss function for evaluating the segmentation model is given as:

(10) $${Loss}_{seg}=1-\frac{\sum_{k=1}^{p}s_{k}\cdot r_{k}}{\sum_{k=1}^{o}s_{k}^{2}+\sum_{k=1}^{p}r_{k}^{2}-\sum_{k=1}^{p}s_{k}\cdot r_{k}}$$

where p indicates the voxel input number; $r_{k}$ indicates the prediction probability and $s_{k}$ indicates the Ground Truth (GT). Furthermore, the focal loss is improvised through optimising the negative and positive sample imbalance and given as:

(11) $${Loss}_{focal}=-\frac{1}{o}\sum_{k=1}^{o}\left[\vartheta s_{k}{\left(1-r_{k}\right)}^{\tau }\log r_{k}+(1-\vartheta )(1-s_{k})r_{k}^{\tau }log(1-r_{k})\right]$$

where α indicates the balancing factor and γ indicates the focusing parameter; $N_{focal}$ is utilized for the segmentation of smaller regions. Thus, the whole segmentation is given as:

(12) $${Loss}_{seg}={Loss}_{jaccard}+{Loss}_{focal}\cdot \rho$$

Furthermore, η denotes the weight factor and the whole segmentation model is given as:

(13) $${Loss}_{SDS}=\sum_{u=2}^{u=3}\varepsilon^{u}\cdot {Loss}_{seg}^{u}+\epsilon^{u}\cdot {Loss}_{seg}^{m}$$

where $\epsilon^{u}$ and $N_{seg}^{m}$ indicates the weight and optimal loss of the sth stage.

(14) $$N_{class}=\sum_{l=1}^{P}\left[s_{l}\log r_{l}+(1-s_{l})log(1-r_{j})\right]$$

(15) $${Loss}_{Integrated\_task\_loss}=\left(\frac{1}{2\vartheta_{1}^{2}}\right)\cdot L_{SDS}+\left(\frac{1}{2\vartheta_{1}^{2}}\right)\cdot {Loss}_{class}+log\vartheta_{1}\vartheta_{2}$$

## 4. Performance Evaluation

LGCNet is designed for brain tumour analysis with the objective of segmentation, identification and grading, which would aid neurologists in diagnosing and reporting the tumour and suggesting the optimal treatment. This section evaluates the proposed model using different metrics. MRI sequences underwent intensity normalization, skull stripping, and resizing. The data were split as 80% training and 20% testing, indicating the use of a representative and balanced dataset that includes both HGG and LGG patients.

The training was carried out on a Tesla P100 GPU with 16 GB of RAM of 300 epochs. The brain tumour segmentation is cropped from the original image width (240 mm) and height (160 mm), while maintaining the linear characteristics and distribution relationship of the image distribution. The training was conducted using the PyTorch v2.0 deep learning framework, leveraging its flexibility and efficiency for model development and experiment, with an Adam optimiser applied to update the model parameters chosen for its adaptive learning rate capabilities and effectiveness in handling sparse gradients. The weight decay was set to 1 × 10^−4^ and employed to prevent any overfitting by penalizing large weights. The initial learning rate was 2 × 10^−4^, providing a balanced starting point for model stability, the batch size was 20, and the number of epochs was set to 300. To avoid overfitting, we employed an early stopping mechanism, where the training automatically stops if the minimum loss stabilizes after 18 iterations.

The proposed model focuses on designing the loss function for different tasks, a hybrid loss function (Dice + focal loss) is used. The Dice loss addresses the overlap accuracy in segmentation, while the focal loss handles class imbalance, which is especially important in small tumour regions. This combined approach allows better optimization for both segmentation and classification.

A hybrid loss function is computed for the segmentation approach, combining multiple loss functions. Ground truth (GT) data, which represent the accurate, labelled segmentations provided by experts, are crucial for training and evaluating the performance of the model.

The evaluation metrics include the sensitivity, specificity, and Hausdorff distance. The sensitivity (recall) measures the proportion of actual positives that are correctly identified by the model, where TP is the number of true positives and where FN is the number of false negatives:

(16) $$\mathrm{Sensitivity}=\frac{TP}{TP+FN}$$

The specificity measures the proportion of actual negatives that are correctly identified by the model:

(17) $$\mathrm{Specificity}=\frac{TN}{TN+FP}$$

The Hausdorff distance is a measure of the maximum distance between the predicted segmentation and the ground truth segmentation. It is used to evaluate the spatial accuracy of the segmentation:

(18) $$H(A,B)=max\{{sup}_{a\in A}{inf}_{b\in B}d(a,b),{sup}_{b\in B}{inf}_{a\in A}d(a,b)\}$$

where A and B are the sets of points in the predicted and ground truth segmentations, respectively, and d (a, b) is the distance between points a and b.

### 4.1. Dataset Details

**Figure 8:**
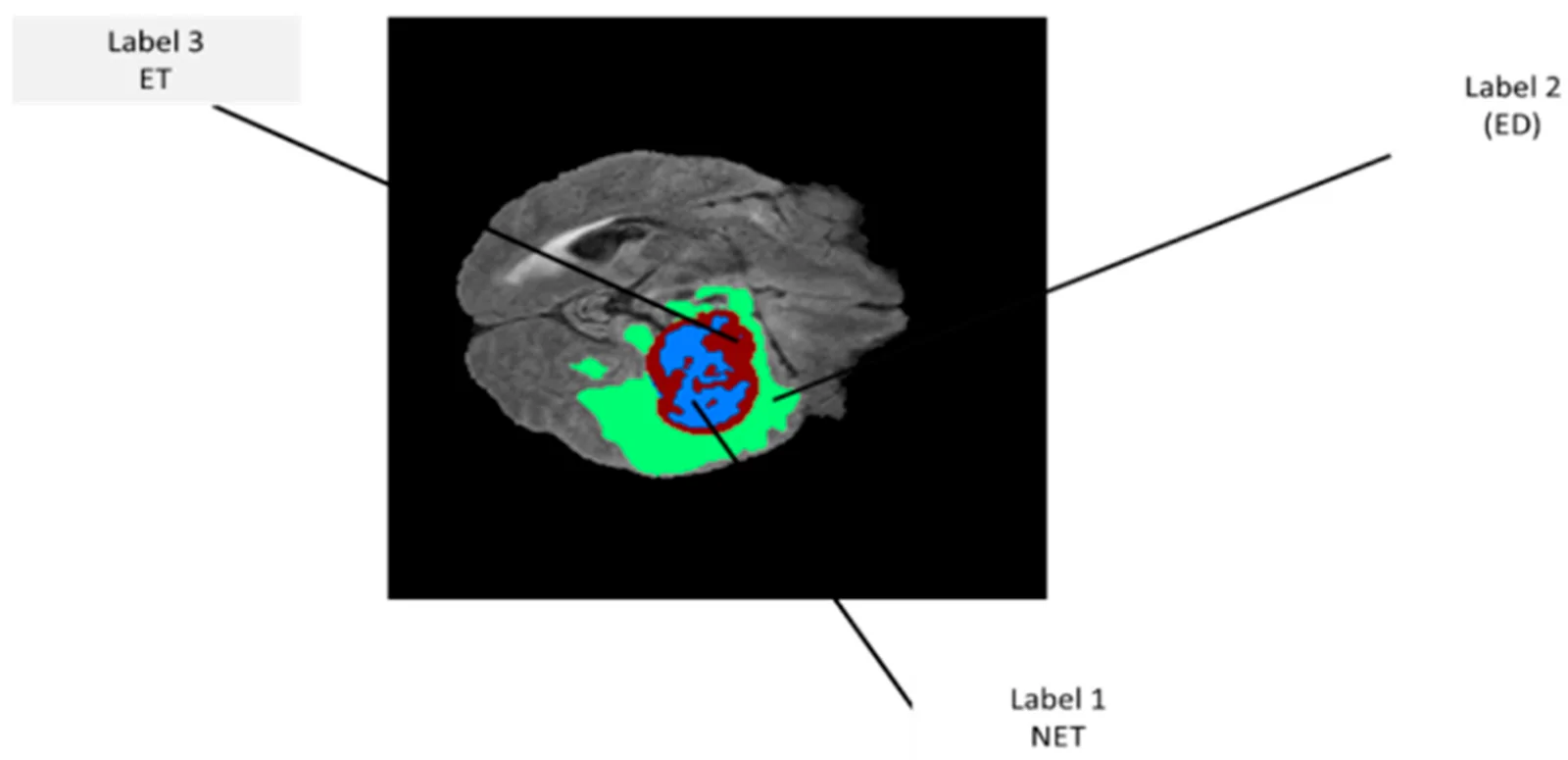
Dataset label visualization.

The proposed model is evaluated by considering the brain MRI dataset of the Multimodal Brain Tumor Segmentation Challenge (BraTS challenge) 2019 [37,38,39]. This dataset comprises various sequences (T1, Gd-enhanced T1, T2 and FLAIR sequences) for patients diagnosed with HGG or LGG. In this study, all four sequences were used, and three datasets were obtained: a training dataset comprising data from 355 patients, a validation dataset comprising data from 125 patients, and a testing dataset comprising data from 167 patients. The training dataset included data from 76 LGG patients and 259 HGG patients and included three GT segmentation labels. The image shown in Figure 8 is a GT image from the BraTS 2019 dataset with the three labels, where Label 1 is a non-enhanced tumour (NET (non-enhanced tumour), Label 2 is an edema, and Label 3 is an enhanced tumour (ET).

### 4.2. Comparison Method

The following approaches are considered for comparison purposes:

- Multi-resolution 3D CNN [40]: Multi-resolution 3D CNN is a deep segmentation approach for glioma detection in 3D in pre-operative patients; thereafter, a classification mechanism based on a random forest algorithm is adopted for survival prediction. This deep architecture for segmentation encompasses two different resolutions using two parallel streamlines. First, a deep CNN is used for learning the local features and the other deep CNN is for local.
- 3D U-Net [41]: This work introduces brain-wise normalization along with a patch-based approach to train the model for segmentation. Thereafter, a network is introduced, which uses the features extracted to predict the survival period of patients after undergoing surgery. The model uses a single GPU platform, which takes a single image as input while training.
- Synthetic segmentation [42]: A framework is designed for synthetic segmentation, which translates a FLAIR MRI into a high-contrast synthetic image; synthesis is carried out on a generative adversarial network that decreases the real channels. Each patient is considered, and several regression mechanisms are utilized for prediction.
- U-Net++ [43]: This model adopts a variation of the U-Net architecture and optimizes loss function, post-processing mechanism, convolution block, deep supervision, and data augmentation. This model tends to present a more lightweight architecture than other U-Net variations. This architecture is considered as the existing model for our research work.
- Semantic segmentation [44]: This work adopts a semantic approach for MRI segmentation; furthermore, an encoder–decoder architecture along with a loss function is developed by considering the challenge dataset.

### 4.3. Visual Comparison

Table 2 presents a visual comparison of the segmentation of the LGG sample, where five different slices are considered. In Table 2, the first row (A) is actual images from the dataset, (B) is segmented ground truth images (C) is existing images segmented using U-Net++, and (D) presents images segmented using the proposed model segmentation. An initial observation shows that the LGCNet model presented in (D) is more accurate than the existing approach (U-Net++). However, it is less accurate due to an imbalance in the datasets.

**Figure 9:**
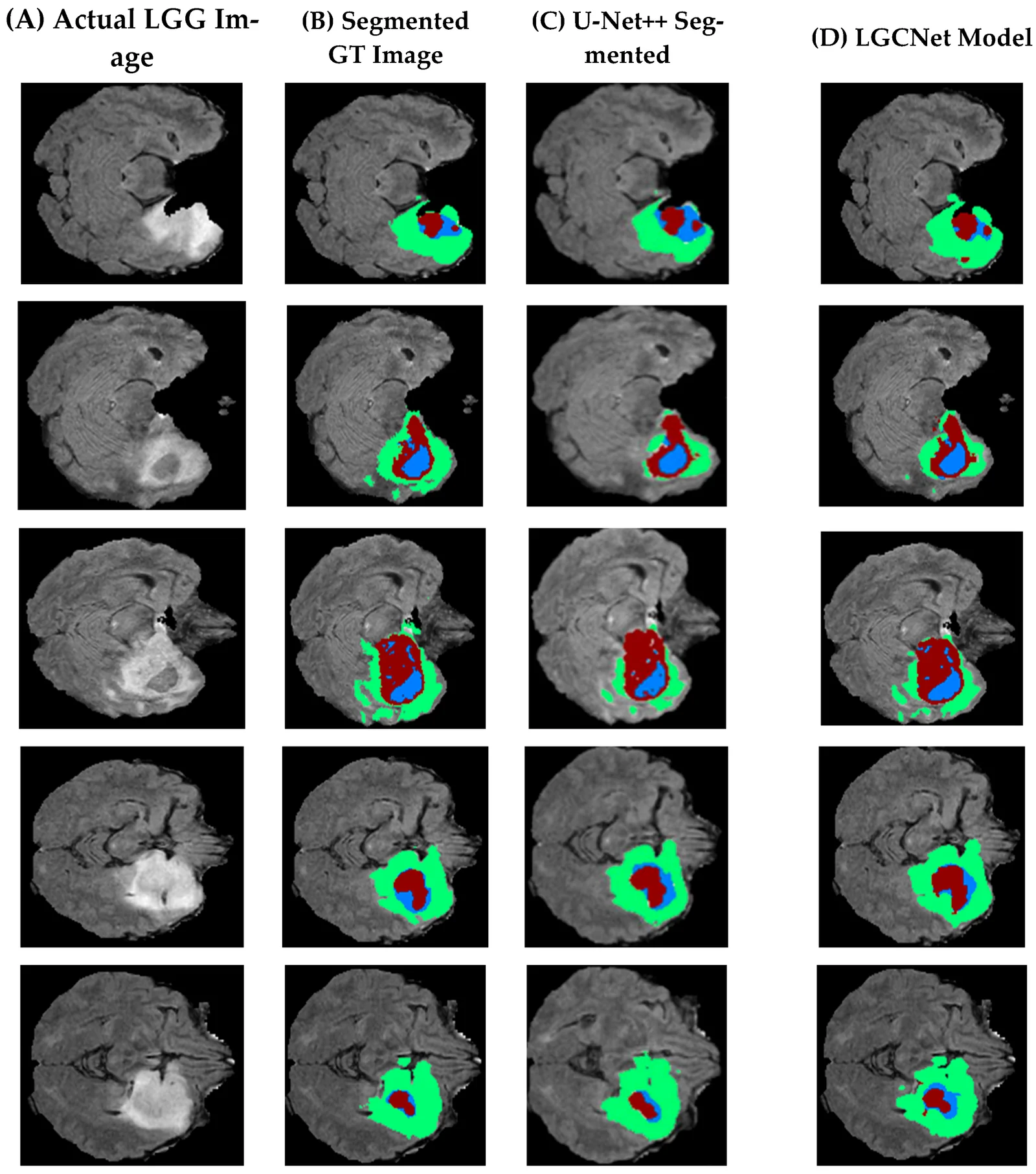
Segmented images of five slices of a brain with an LGG.

Figure 9 presents the segmentation of five slices of a brain with an HGG, where Column A shows an actual HGG image, Column B shows a segmented ground truth image, Column C shows an image segmented using U-Net++, and Column D shows an image segmented by the proposed model. 

**Figure 10:**
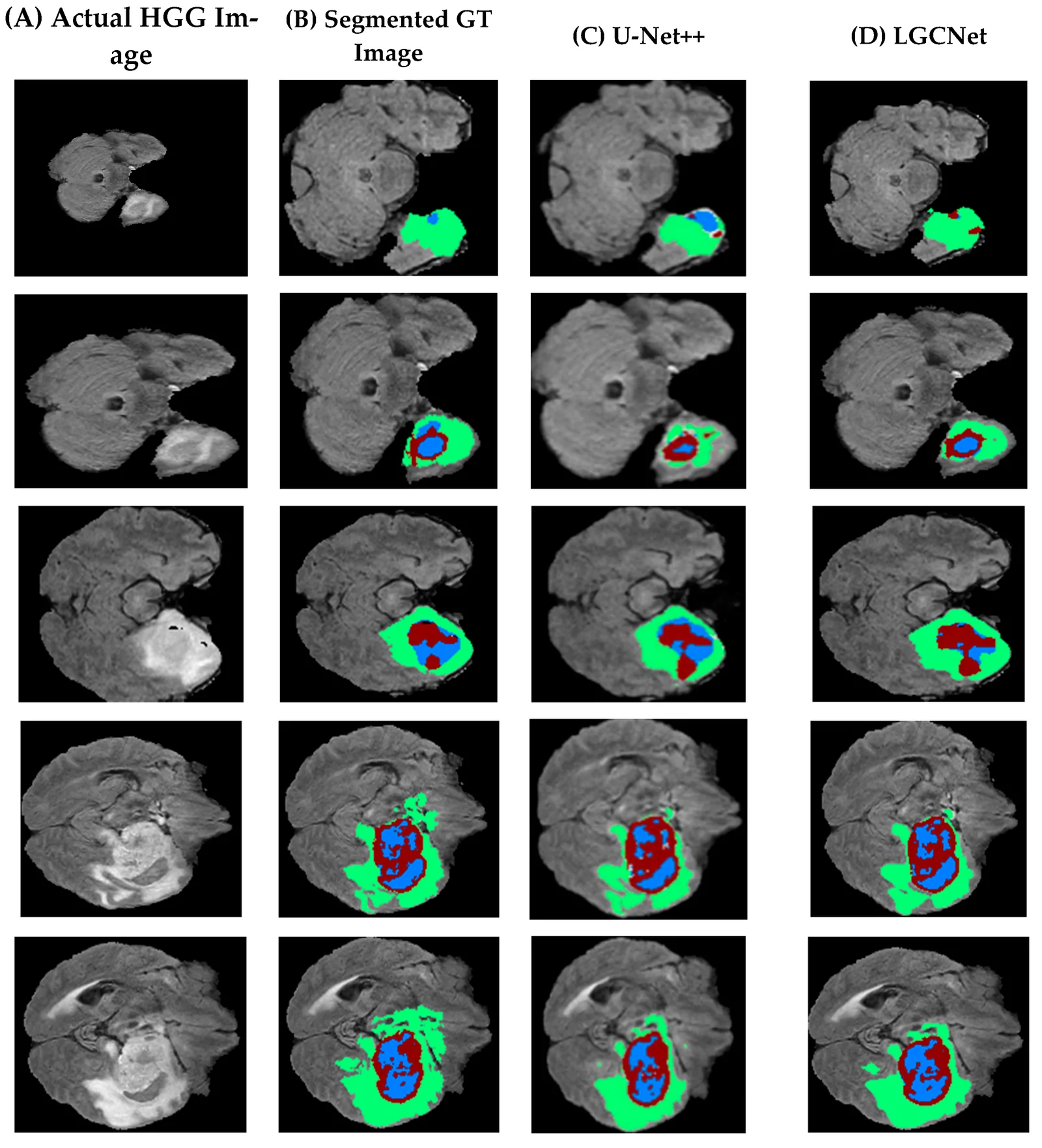
Segmented images of five slices of a brain with an HGG.

By comparing this figure to Figure 8, it can be noticed that the HGG classification is more accurate than the LGG and that the labels in LGCNet are better than those in the existing model. Moreover, it can be observed that the proposed model is able to identify and segment all three labels (Label 2, Label 3 and label more optimal than the existing model), as shown in Figure 9 and Figure 10.

### 4.4. Evaluation Criteria and Performance Analysis

This section presents a comparison of the various models in terms of the sensitivity, specificity, Dice score and Hausdorff distance to evaluate and prove the efficiency of the proposed model. This research considers three classes for evaluation—whole tumour (WT), enhanced tumour (ET) and tumour core (TC)—as well as the Hausdorff distance. The results for each are discussed below.

#### 4.4.1. Sensitivity

**Figure 11:**
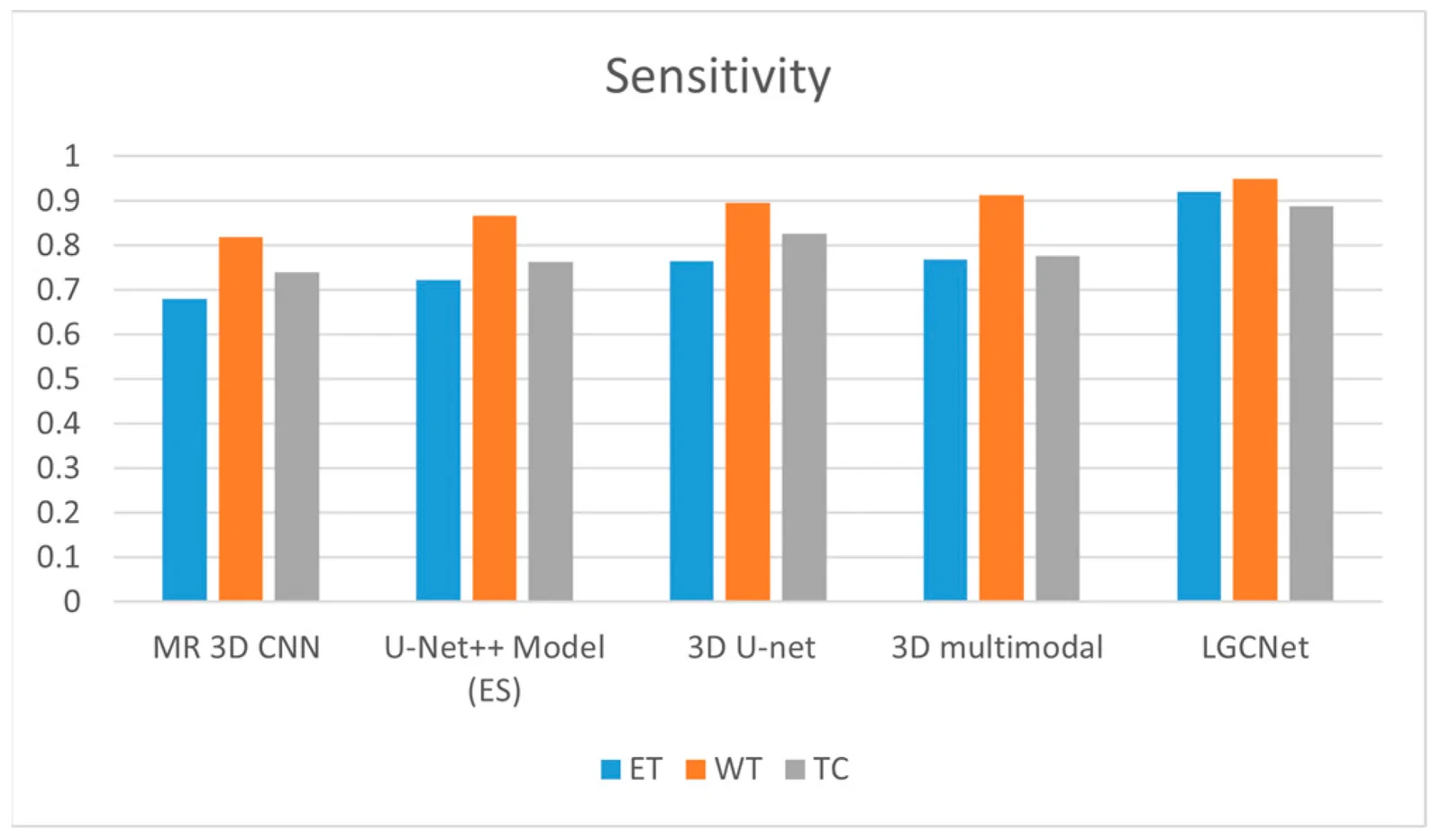
Sensitivity comparison of the three classes.

The sensitivity is defined as the model’s ability to designate a tumour as a true positive, where 1 indicates perfect sensitivity and where 0.5 indicates a random draw. High sensitivity indicates that there are fewer false negative outcomes and that fewer cases have been missed. Figure 11 and Table 2 present a comparison for the three labels (ET, WT and TC) when using state-of-the-art techniques. In the case of ETA, LGCNet has higher sensitivity (0.922) than the 3D multimodal technique (0.766); for NTA, the sensitivity of the LGCNet is 0.95, which is slightly higher than the value of 0.913 for the 3D multimodal. For NTA, LGCNet achieves a value of 0.888, which is higher than that of 3D U-Net (0.826). Moreover, other state-of-the-art approaches such as multi-resolution (MR) 3D CNN, 3D U-Net and the existing U-Net++ model achieve a maximum metric value of 0.76 for the ET class, which is below the value of 0.9 for the WT class. However, when considering the CT class, the 3D U-Net achieves a higher sensitivity value than the existing approach.

#### 4.4.2. Specificity

The specificity is defined as the ability of the model to designate a particular MRI image that does not show a tumour as negative. A specificity of one indicates that the model has a 0% false positive rate.

**Figure 12:**
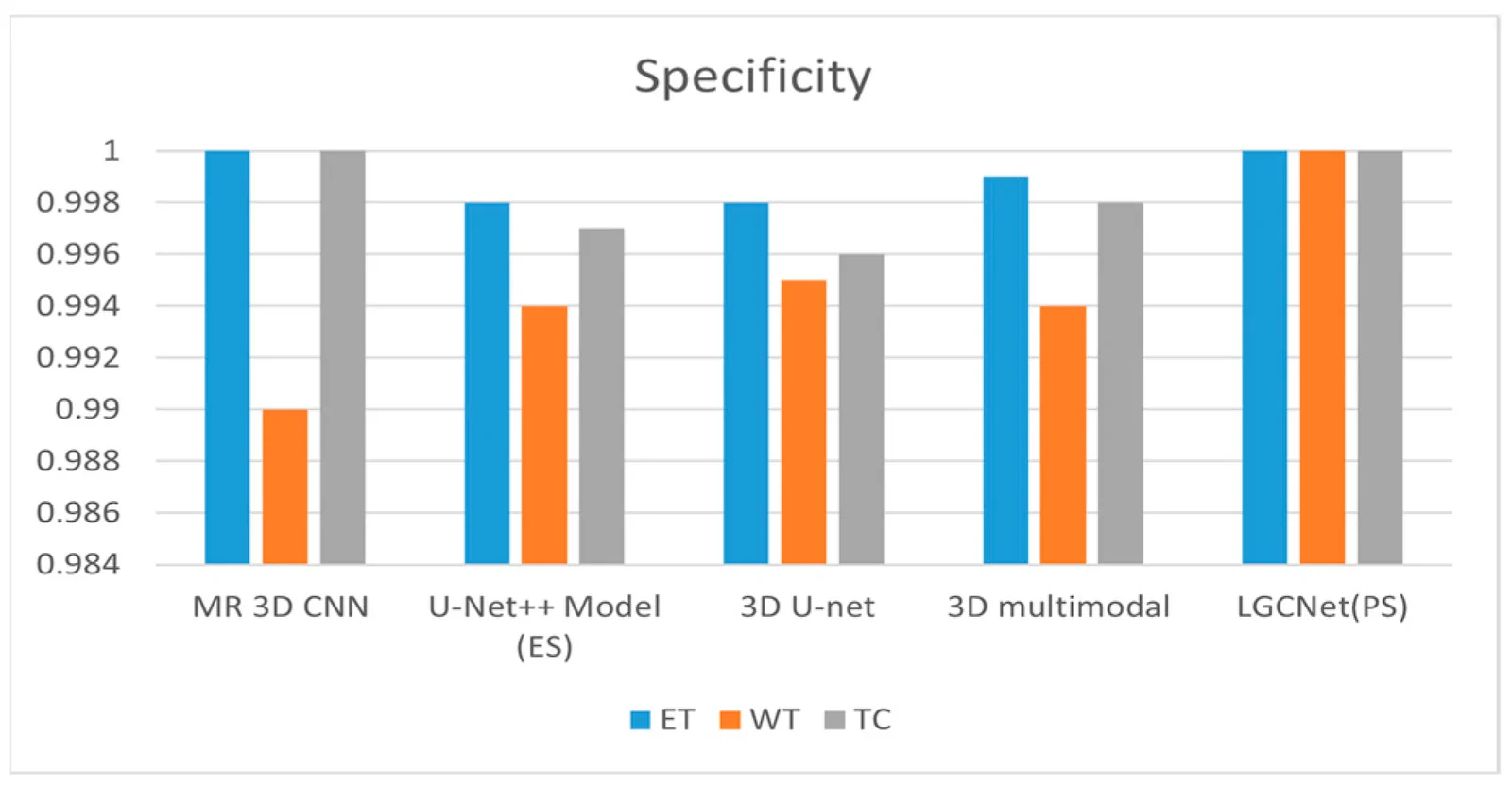
Specificities for three labels for different models.

Figure 12 and Table 3 show the specificity of various models as compared to the proposed model. The proposed model shows a specificity of one for all three labels. For ET, the multi-resolution 3D CNN and the proposed LGCNet both achieve a specificity of 1; for WT, 3D U-Net only achieves a specificity of 0.995. For TC, both the multi-resolution 3D U-Net and the LGCNet model achieve a specificity of one.

#### 4.4.3. Dice Score

The Dice score is an indicator of the similarity of two datasets. For image segmentation, the score is computed as the proportion of overlap of the segmented images normalized by the total size of the images, where a score of zero indicates no overlap and a score of one indicates complete overlap. The Dice score (*DSC*) is calculated as:

(19) $$DSC=2\frac{TP}{2(TP+FN+FP)}$$

where TP indicates a true positive, *FP* indicates a false positive, and *FN* indicates a false negative.

**Figure 13:**
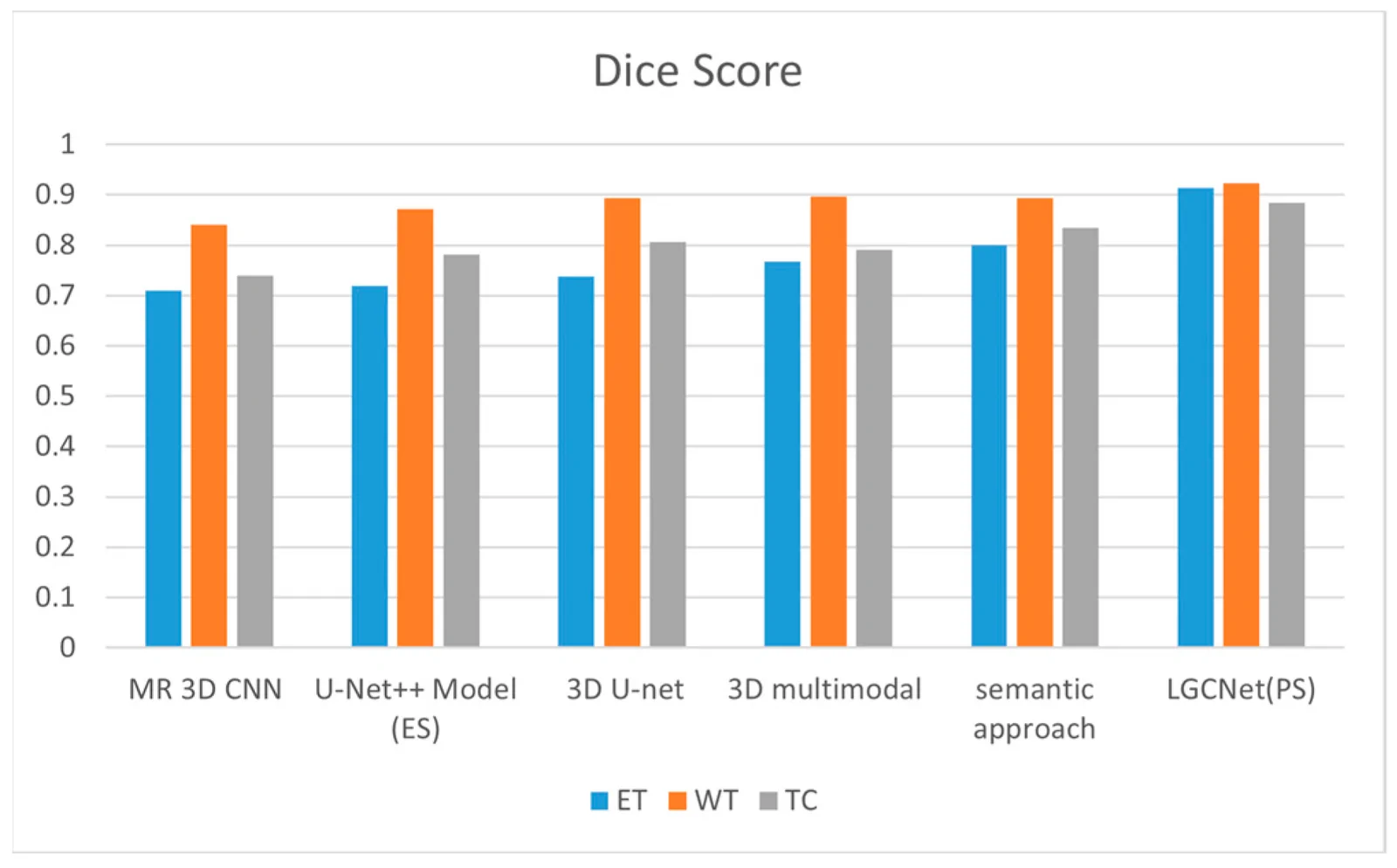
Dice scores for the different segmentation methods.

Figure 13 and Table 4 show the Dice scores for the different segmentation methods for all three labels. For the ET class, the best-performing models are the semantic approach (with a value of 0.8) and the LGCNet model (with a value of 0.913). For the WT class, the LGCNet model achieves a Dice score of 0.923, while the 3D U-Net model has a score of 0.897. For the ET class, the 3D multimodal achieves a score of 0.767, while the LGCNet model has a score of 0.913, while the other three models (MR 3D CNN, U-Net++ and 3D U-net) all have scores below 0.75.

#### 4.4.4. Hausdorff Distance

The Hausdorff distance is a performance metric that is widely utilized for measuring the distance between two point sets. Here, it is utilized to compare the GT (Ground Truth image to the segmentation predicted results to enable a ranking of the various segmentation results.

**Figure 14:**
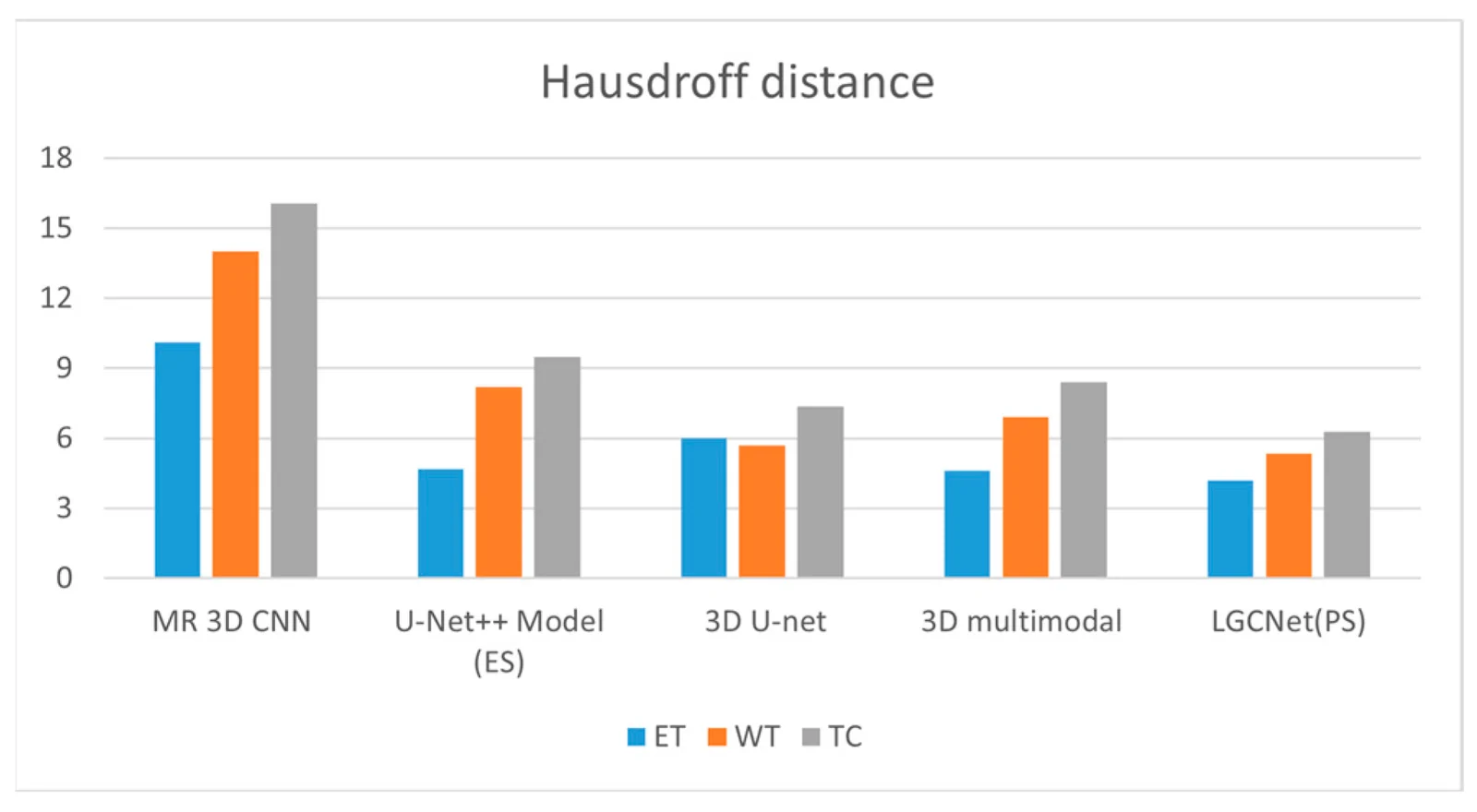
Hausdorff distances for the different segmentation methods.

Figure 14 and Table 5 show the Hausdorff distances of the proposed model and existing models for all three labels. For ET, the 3D multimodal LGCNet achieves a distance of 4.5, while the 3D multimodal model has a distance of 4.6. For WT, the 3D multimodal model has a distance of 6.9, while the LGCNet model has a distance of 5.8. For TC, the 3D U-Net model has a distance of 7.357, while the LGCNet model has a distance of 6.273.

### 4.5. Comparative Analysis and Discussion

This section discusses the improvement of the LGCNet model over existing models. LGCNet assumes that the attention network improves the model performance and the metric value. The 3D approach is one of the most successful models, but it has several drawbacks, such as feature extraction at a user-defined level and the lack of a dedicated network for a task, which makes it more vulnerable. LGCNet uses a dedicated network for a specific task and is compared with the 3D approach; for analysis, U-Net++ is considered as an existing model in this research. For the Dice coefficient metrics, the semantic approach achieves the highest value, while LGCNet achieves 14.12%, 3.24% and 5.95% improvement over the U-Net++ model for ET, WT and TC, respectively. For the Hausdorff distance metric, LGCNet also achieves an improvement of 8.69%, 49.27% and 25.32% for these labels. LGCNet achieves an improvement of 19.896%, 4.05% and 14.285%, respectively, in sensitivity over the other best model. Finally, LGCNet achieves a metric value of 100%, which is identical to the value of another model.

The LGCNet model effectively represents different tumour grades and sizes through its dual-network architecture, which integrates local context attention (LCANet) and global context attention (GCANet) to capture detailed local features and broader contextual information. This design enhances the model’s ability to accurately segment and grade various tumour grades from subtle low-grade gliomas (LGGs) to aggressive high-grade gliomas (HGGs) as well as to handle tumours of different sizes. Small tumours, which often have less distinct boundaries, are highlighted through the attention mechanisms, while larger tumours are captured with high-resolution features that preserve both the overall structure and the local details. However, the model’s generalisability to other datasets is influenced by the variability of tumour morphology and imaging conditions, which can differ significantly across datasets. The dependency on high-quality annotated data and the risk of overfitting to specific characteristics of the training data pose challenges. Additionally, while LGCNet is tailored for multimodal MRI images, applying it to datasets with different imaging modalities or characteristics may require significant modifications, potentially affecting its performance. Thus, while LGCNet is robust within its domain, its effectiveness on other datasets depends on several factors, including the data similarity, annotation quality, and adaptability to different imaging conditions.

While the LGCNet model for brain tumour segmentation and grading presents significant advancements, it also has notable drawbacks. The model’s computational complexity and high memory requirements necessitate powerful hardware, limiting its accessibility. It is highly sensitive to hyperparameters, making optimal tuning time-consuming. There is also a risk of overfitting, especially with limited annotated data, which the model heavily depends on. Implementing LGCNet involves complex components, which may pose challenges for some researchers. Its architecture, tailored for brain MRI images, may not generalize well to other medical imaging tasks. Additionally, the “black box” nature of deep learning models such as LGCNet limits their interpretability, which is crucial for clinical trust and adoption.

The LGCNet model’s dual-task architecture allows for simultaneous segmentation and grading of gliomas, reducing the time and complexity of radiological workflows. With real-time inference capability, the model is suitable for direct integration into clinical systems such as PACSs (Picture Archiving and Communication Systems). By producing immediate and interpretable outputs, LGCNet can assist radiologists in identifying tumour boundaries and assigning accurate glioma grades, thereby acting as a decision-support tool. Its modular structure allows it to be customized and extended to fit different imaging protocols and clinical software environments.

## 5. Conclusions

The human brain is an intriguing model whose complexity requires sophisticated means for understanding and characterizing behaviour. Thus, brain tumour detection and classification has proven to be an onerous task, even considering advanced MRI techniques, due to the similarity between the tumour cells and the cells in the surrounding tissue. This research presents a new model, the LGCNet model, for detection and analysis with a dual attention network (a local context-aware network and a global context attention network) for the segmentation and grading of brain tumours. The global context attention network extracts the universal features, whereas the local attention network is concerned with task-based features. Both are integrated into the proposed model, and the loss functions are combined for both segmentation and classification. LGCNet is evaluated considering the three labels (ET, WT and TC) with two different grading levels (HGG and LGG) for images in the BraTS 2019 dataset. A performance evaluation is carried out with different metrics, such as the sensitivity, specificity, Dice score, and Hausdorff distance. Both a visual comparison and a comparison of the performance metrics are conducted. The findings indicate that the LGCNet model possesses a research scope with a dedicated network, and the unrivalled learning capability of the DL-based model has made it the standard choice for detection and classification. Various opportunities remain for future explorations of segmentation and classification, beginning with pre-processing and post-processing. Furthermore, other improvements can include a further reduction of overfitting with the data augmentation technique.

## Figures and Tables

**Table 1:** Summary of Existing Models.

Model	Key Features	Advantages	Limitations
ResNet50 [23]	Using a deep residual network to extract features	Strong performance on classification tasks	Limited ability to segment
BrainMRNet [24]	Attention-based CNN for tumour detection	Improves focus on tumour region	Task-specific, not multi-purpose
FusionModel [25]	Combines U-Net and Transformer	Captures long-range dependencies	High training complexity
MMD-VAE [26]	Multimodal variational autoencoder	Combining many MRI modalities	Complex architecture, needs large datasets
CANet [27]	Context-aware network using attention + CRFs	Use of conditional information and good contextual learning	Overfitting risk and intricate post-processing

**Table 2:** Specificity of different models.

	ET	WT	TC
**MR 3D CNN**	0.68	0.82	0.74
**U-Net++ model (ES)**	0.723	0.867	0.763
**3D U-Net**	0.766	0.897	0.826
**3D multimodal**	0.769	0.913	0.777
**LGCNet**	0.922	0.95	0.888

**Table 3:** Sensitivity of different models.

	ET	WT	TC
**MR 3D CNN**	1	0.99	1
**U-Net++ model (ES)**	0.998	0.994	0.997
**3D U-Net**	0.998	0.995	0.996
**3D multimodal**	0.999	0.994	0.998
**LGCNet (PS)**	1	1	1

**Table 4:** Dice scores for the tumour segmentation models.

	ET	WT	TC
**MR 3D CNN**	0.71	0.84	0.74
**U-Net++ model (ES)**	0.719	0.871	0.782
**3D U-Net**	0.737	0.894	0.807
**3D multimodal**	0.767	0.897	0.79
**Semantic approach**	0.8	0.894	0.834
**LGCNet (PS)**	0.913	0.923	0.884

**Table 5:** Hausdorff distances across the models.

	ET	WT	TC
**MR 3D CNN**	10.11	14	16.06
**U-Net++ Model (ES)**	4.6868	8.216	9.475
**3D U-Net**	5.994	5.677	7.357
**3D multimodal**	4.6	6.9	8.4
**LGCNet (PS)**	4.2	5.35	6.273

## Data Availability

The original contributions presented in this study are included in the article. Further inquiries can be directed to the corresponding authors.
